# Characterising spatial patterns of neglected tropical disease transmission using integrated sero-surveillance in Northern Ghana

**DOI:** 10.1371/journal.pntd.0010227

**Published:** 2022-03-08

**Authors:** Kimberly M. Fornace, Laura Senyonjo, Diana L. Martin, Sarah Gwyn, Elena Schmidt, David Agyemang, Benjamin Marfo, James Addy, Ernest Mensah, Anthony W. Solomon, Robin Bailey, Chris J. Drakeley, Rachel L. Pullan

**Affiliations:** 1 Faculty of Infectious and Tropical Diseases, London School of Hygiene & Tropical Medicine, London, United Kingdom; 2 Institute of Biodiversity, Animal Health and Comparative Medicine, University of Glasgow, Glasgow, United Kingdom; 3 Research Team, Sightsavers UK, Haywards Heath, United Kingdom; 4 Division of Parasitic Diseases and Malaria, Centers for Disease Control and Prevention, Atlanta, Georgia, United States of America; 5 Ghana Office, Sightsavers, Accra, Ghana; 6 Neglected Tropical Disease Team, Ghana Health Service, Accra, Ghana; 7 FHI 360, Accra, Ghana; 8 Department of Control of Neglected Tropical Diseases, World Health Organization, Geneva, Switzerland; The University of Hong Kong, CHINA

## Abstract

**Background:**

As prevalence decreases in pre-elimination settings, identifying the spatial distribution of remaining infections to target control measures becomes increasingly challenging. By measuring multiple antibody responses indicative of past exposure to different pathogens, integrated serological surveys enable simultaneous characterisation of residual transmission of multiple pathogens.

**Methodology/Principal findings:**

Here, we combine integrated serological surveys with geostatistical modelling and remote sensing-derived environmental data to estimate the spatial distribution of exposure to multiple diseases in children in Northern Ghana. The study utilised the trachoma surveillance survey platform (cross-sectional two-stage cluster-sampled surveys) to collect information on additional identified diseases at different stages of elimination with minimal additional cost. Geostatistical modelling of serological data allowed identification of areas with high probabilities of recent exposure to diseases of interest, including areas previously unknown to control programmes. We additionally demonstrate how serological surveys can be used to identify areas with exposure to multiple diseases and to prioritise areas with high uncertainty for future surveys. Modelled estimates of cluster-level prevalence were strongly correlated with more operationally feasible metrics of antibody responses.

**Conclusions/Significance:**

This study demonstrates the potential of integrated serological surveillance to characterise spatial distributions of exposure to multiple pathogens in low transmission and elimination settings when the probability of detecting infections is low.

## Author summary

Following implementation of successful interventions, one of the primary challenges for neglected tropical disease programmes is identifying areas with remaining disease transmission. As disease prevalence decreases, these infections become increasingly rare and hard to detect. Serological assays measure long-lived disease-specific antibody responses indicating past exposure to pathogens and increase the probability of detecting disease transmission. Here, we integrate serological assays with environmental and spatial data to map priority areas for surveillance for multiple neglected tropical diseases in Northern Ghana using the two-stage cluster-based survey platform established for trachoma surveillance. The use of multiplex bead assays measuring exposure to multiple pathogens allows integrated surveillance of diseases of interest to the national control programme. We identify areas with high risks of transmission to selected diseases as well as areas with high uncertainty which are priorities for future control and surveillance efforts. Together, this highlights the utility of multiplex serological platforms as a tool for integrated surveillance and mapping of neglected tropical diseases.

## Introduction

Neglected tropical diseases (NTDs), such as trachoma, schistosomiasis, onchocerciasis, lymphatic filariasis and soil-transmitted helminthiases, cause substantial public health burdens globally. With increasing investment in NTD control, elimination and eradication programmes, community-based surveys are used to monitor impacts of interventions, identify residual transmission and target high-risk populations [1,2]. These data are frequently analysed using geostatistical models, relating infection metrics to environmental and spatial covariates to define the geographical extent of transmission, predict disease burdens and prioritise areas of uncertainty [3,4]. However, as countries move towards elimination, infections become increasingly rare and difficult to monitor through community-based surveys. Such challenges are currently faced by NTD control programmes operating in Northern Ghana where trachoma elimination was achieved in 2018 and only isolated transmission is reported for onchocerciasis and other NTDs [5,6]. New approaches are needed to cost-effectively identify remaining infections and areas at high risk of recrudescence.

As transmission of infectious diseases decrease, the probability of detecting infections becomes correspondingly low and requires prohibitively large sample sizes to identify infections. For many diseases, this also corresponds with more pronounced spatial heterogeneity, with transmission concentrated in specific geographic areas or sub-populations [7–9]. Serological assays are potentially sensitive tools in these contexts; by measuring specific antibody responses reflecting previous exposure to pathogens, changes in transmission can be detected over longer durations in higher proportions of populations [10]. This enables estimation of the force of infection and historical transmission intensities using age-stratified antibody magnitudes or longitudinal sampling, allowing exploration of control measure impacts over time [11–14]. These sero-epidemiological methods have been applied to NTDs including trachoma, lymphatic filariasis, schistosomiasis, and onchocerciasis as well as enteropathogens and are strongly correlated with commonly used metrics of infection prevalence (e.g. [13,15–17]).

The development of multiplex serological assays increases the operational feasibility of serological techniques by enabling measurement of a broad range of responses with high repeatability from limited blood samples. Multiplex bead assays (MBA) are a well-validated method to measure population-level serological responses to a wide panel of NTDs, malaria, vaccine preventable diseases and other infections [18]. These assays allow measurement of antibody responses to multiple pathogens simultaneously for relatively low cost, creating new opportunities for integrated serological surveillance and maximising the benefit of limited resources [19,20]. Applying these integrated approaches can detect populations at high risks of multiple infections, identifying public health gaps and allowing targeting of coordinated interventions. Further, analysis of these data within geostatistical modelling frameworks provides new opportunities to characterise the spatial distribution of multiple diseases and identify environmental factors driving residual transmission [21]. Geostatistical modelling typically also allows greater precision of estimates using fewer data points than conventional analytical approaches [22].

Despite increasing collection of serological data, challenges remain in translating these data into actionable programmatic information [23]. Spatial analysis of serological data is complicated by the duration of antibody responses and frequency of exposure to infection; while it is possible to map seropositivity, resulting maps may reflect historical exposure rather than current transmission and have limited utility to programmes. Additionally, applying commonly used geostatistical modelling approaches to quantitative antibody responses may not capture outlying values of high responders, likely representing individuals recently or repeatedly exposed to a pathogen. A recent study demonstrated how the force of infection calculated from paired serological surveys could be used to quantify spatial heterogeneity of schistosomiasis transmission, showing close correlation with other routinely collected measures of schistosomiasis infection intensity [24]. While highlighting the utility of serological assays to characterise spatial patterns of transmission, this modelling approach utilises surveys of the same area over multiple time points to quantify transmission over time, data not routinely collected by NTD programmes. Alternatively, for diseases such as malaria and cholera, detailed longitudinal cohort studies have identified markers of recent exposure or been used to train machine learning approaches to estimate sero-incidence; however, these cohort data are not available for most NTDs [25,26].

Here, we adapt a commonly used Gaussian mixture model and binomial geostatistical models to describe the spatial distribution of antibody responses to multiple diseases in a population-based survey of children in Northern Ghana. Conducted as part of routine surveillance for trachoma elimination, this survey utilised a two-stage cluster-sampling population-based survey design and identified very low levels of the sign “trachomatous inflammation—follicular”, a finding supported by estimates of seroconversion rates [27–29]. Although this low prevalence precludes modelling the spatial distribution of infection, geostatistical modelling of serological data allows prioritisation of geographical areas of potential risk of recrudescence that can be targeted for future trachoma post-validation surveillance efforts. As this survey used a systematically- and randomly- sampled population-based approach rather than purposeful sampling, assessment of spatial patterns of other disease transmission is possible. Using multiplex serological data, we demonstrate how an integrated surveillance approach can be used to estimate the geographical distribution of exposure to other pathogens within this population and identify areas with previously unknown elevated risks of multiple diseases requiring prioritisation for national surveillance programmes.

## Methods

### Ethics statement

This study was approved by the Ghana Health Service Ethics Review Committee (GHS-ERC: 03/07/15) and the London School of Hygiene & Tropical Medicine (10285). Written informed consent was sought from parents or guardians of all participating children. Verbal assent was additionally obtained from children who were able to provide this. The CDC investigators were not considered to be engaged in human subjects research.

### Survey and laboratory methods

**Fig 1:**
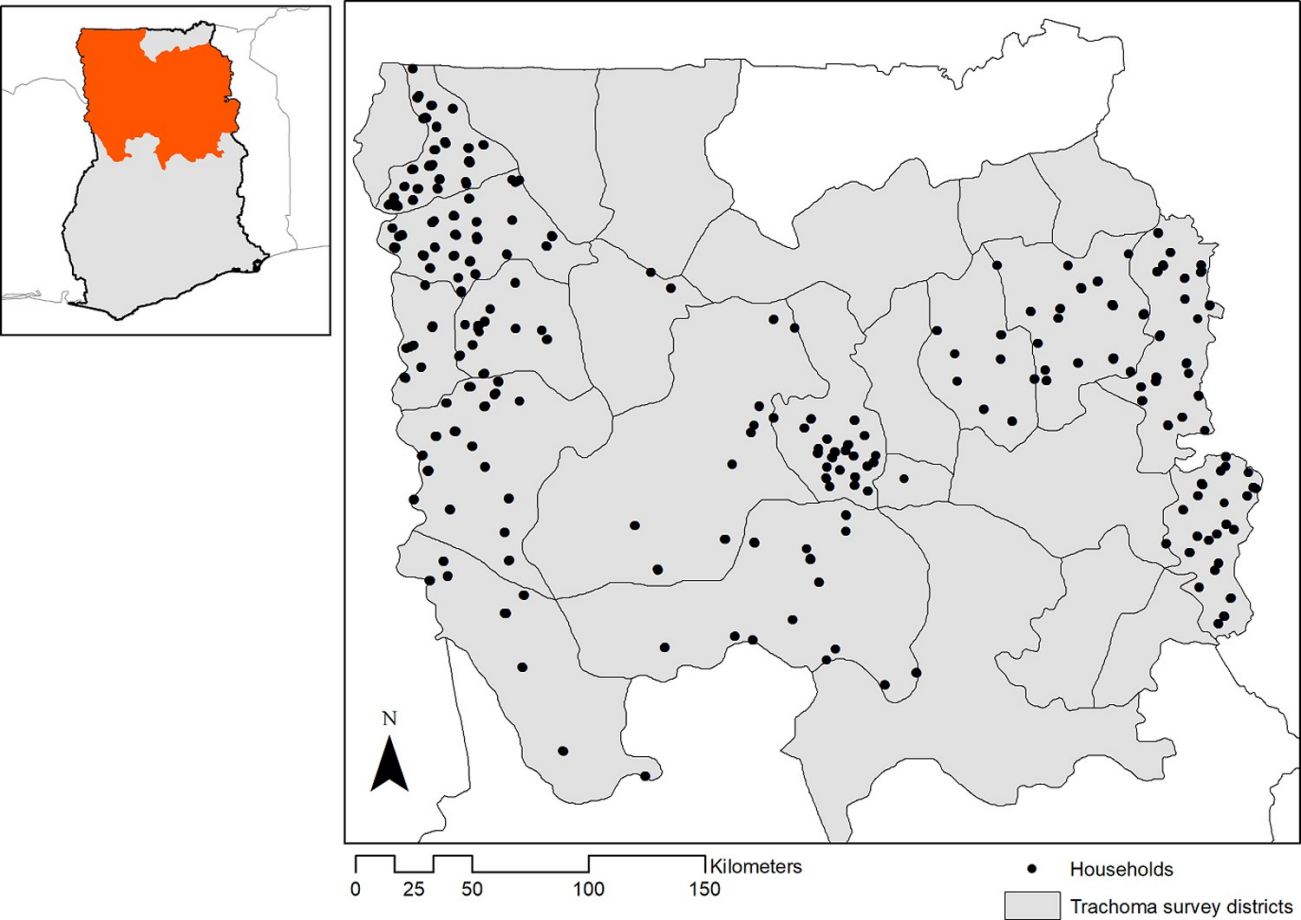
Study site in Northern Ghana; administrative shapefiles obtained from National Information Technology Agency (NITA), Government of Ghana (https://data.gov.gh/dataset/shapefiles-all-districts-ghana-170-districts). The boundaries and names shown and the designations used on this map do not imply the expression of any opinion whatsoever on the part of the authors, or the institutions with which they are affiliated, concerning the legal status of any country, territory, city or area or of its authorities, or concerning the delimitation of its frontiers or boundaries.

This study was conducted in the Northern, North East, Savanna and Upper West regions of Ghana in a predominantly rural agricultural population (Fig 1). The climate is tropical and elevations range from sea level to 900 metres above sea level. This area includes several national parks and multiple rivers, including tributaries to the Volta [27]. From 2015–2016, two-stage cluster-sampled population-based surveys were conducted as part of trachoma elimination pre-validation activities [27]. Briefly, clusters (villages) were sampled with probability proportional to size and households selected using compact segment sampling across 18 evaluation units. All children aged 1–9 years were eligible for inclusion. These ages were targeted as younger children have the highest risk of active trachoma and the primary purpose of the survey was to provide evidence for the validation of elimination of trachoma in Ghana. Basic demographic data and GPS coordinates of household locations were collected electronically using Open Data Kit (www.getodk.org).

All children from consenting households provided a finger-prick blood sample, which was collected and stored on filter paper (Trop-Bio, Townsville, Australia) and processed at the U.S. Centers for Disease Control and Prevention as previously described [28]. Using a multiplex bead assay (MBA), immunoglobulin G antibody responses were measured to the following antigens: Pgp3 trachoma (*Chlamydia trachomatis*), Wb123 lymphatic filariasis (*Wucheria bancrofti*), Ov16 onchocerciasis (*Onchocerca volvulus*), NIE strongyloidiasis (*Strongyloides stercoralis*), soluble egg antigen (SEA) schistosomiasis (*Schistosoma mansoni*), rp17 and TmpA yaws (*Treponema pallidum*), VSP3 giardiasis (*Giardia lamblia*). Glutathione s-transferase (GST) was used as a negative control. MBA were conducted using standard methods [28]. Antibody responses were quantified as median fluorescence intensity (MFI) for each antigen and sample. We excluded samples that had an MFI value for GST over 1000 (3 samples excluded, median GST MFI = 4, IQR: 2–7), which indicates high background levels and unreliable measurements.

### Classification of seropositivity

In the absence of standard controls or clinically characterised sera to determine cut-off values, Gaussian mixture models are commonly used to determine seropositivity, modelling antibody responses as latent populations for a user-defined number of distributions. These distributions can then be used to define antigen-specific cut-off values dependent on the number of observations and ranges of values. While these algorithms are frequently applied to differentiate seronegative and seropositive populations, this approach can be extended to include more than two components, with components corresponding to different degrees of pathogen exposure [30]. As programmes require data on the distribution of recent exposure operationally, and environmental and spatial data reflected current conditions, we fit a three-component Gaussian mixture model to log-transformed antibody data. We assumed these components corresponded to unexposed, historically exposed and recently or repeatedly exposed populations [31]. While antibody responses may have differing numbers of components, we chose to use a three-component mixture model specifically to identify individuals with the highest MFI responses likely to be recently exposed. Other approaches, such as modelling continuous antibody response data, may characterise mean antibody response levels but are unlikely to accurately capture outliers. Similarly, approaches using a predefined centile of the highest responders may identify the individuals with highest likelihoods of recent exposure but cannot be used to assess prevalence as the percentage positive is predetermined [32]. Using an approach described for enteropathogens, where high intensity and repeated exposure is observed in young children, we fit mixture models to children aged under 3 years to ensure sufficient proportions of unexposed individuals that would enable characterisation of the seronegative population [15]. Mixture models were informed by priors on the expected proportions of individuals in each component based on published data and consultations with the NTD programme (Table A in S1 Text). For example, we assumed a low prior probability of recent or historical yaws due to previous reports of elimination in this area [33]. For geostatistical modelling, we defined seropositive individuals as individuals belonging the highest component and most likely to have recent or repeated exposure.

### Environmental and spatial data

We assembled plausible spatial and environmental covariates including population density [34], travel time to the nearest city [35], insecticide treated bednet coverage, soil types [36], forest cover [37] and bioclimatic factors representing long-term and more recent historical annual trends, seasonality and extreme or limiting environmental factors [38]. Topographic wetness index was calculated from a digital elevation model [39] and Euclidean distance to roads and water bodies were calculated at 30m resolution from Open Street Map data [40] (Table A in S1 Text). Normalised difference vegetation index and normalised difference water index were calculated from cloud-free composites of Landsat data during the survey time period [41]. Pearson correlation analysis was used to assess multicollinearity between variables, excluding variables with correlation coefficients > 0.75. Models were fit separately to assess exposure to each pathogen and covariates were selected based on the Bayesian information criteria (BIC) of the most parsimonious non-spatial binomial regression model for each disease and residual spatial autocorrelation was assessed using Moran’s I. To generate covariates for predictions, we resampled all data to 1000m resolution and extracted values. We excluded all national parks and protected areas [42].

### Geostatistical modelling

Final models were developed within a Bayesian hierarchical modelling framework. In addition to including identified covariates, these models also used a geostatistical modelling approach to account for residual spatial autocorrelation not explained by these covariates. For each disease, we fit separate models with *p*(*x_i_*) denoting the seroprevalence at locations *x_i_*, *i* = 1…*n*, with *m_i_* individuals sampled per location. The full model was specified as:

$$Y_{i}∼Binomial(m_{i},{p(x}_{i}))$$

With the linear predictor for the binomial model specified as:

$$logit({p(x}_{i}))=\beta_{0}+{d(x}_{i})\prime \beta +w_{i}$$

Where *β*_0_ represents the intercept, ***d***(***x_i_***)′***β*** represents a vector of location specific covariate effects and *w_i_* represents the spatial effect. Weakly informative priors of Normal (0, 100) were used for intercepts and fixed effect coefficients and penalised complexity priors were used for the spatial effect [43]. As Moran’s I showed significant spatial autocorrelation for all diseases modelled, we additionally fit models with the spatial effect modelled as a Matern covariance function between locations *s*_*j*_ and *s*_*k*_:

W∼MultivariateNormal(0,Σ)

$$\Sigma_{jk}=Cov(\xi (s_{j}),\xi (s_{k}))=Cov(\xi_{j},\xi_{k})=\frac{\sigma^{2}}{{\Gamma (\lambda )2}^{\lambda -1}}{\left(\kappa ||s_{i}-s_{k}||\right)}^{\lambda }K_{\lambda }(\kappa ||s_{j}-s_{k}||)$$

Final models were assessed using the deviance information criteria (DIC) and conditional predictive ordinate (CPO). All models were implemented in R statistical software version 3.6 using Integrated Nested Laplace Approximation (INLA), using 1,000 samples to estimate posterior probabilities [44].

To prioritise areas for future sampling, we calculated exceedance probabilities for a threshold of 10% seroprevalence of high responders. We chose a threshold of 10% as this indicates on-going transmission in most settings, although this threshold could be adjusted based on programmatic requirements. These exceedance probabilities represent the probability that an area has over 10% seroprevalence, enabling visualisation of high-risk areas as well as identification of areas with high uncertainty [45]. Priority areas with high probabilities of multiple diseases were identified by overlaying classified exceedance probabilities. To define areas with high probabilities of recent exposure, we classified high-risk areas as areas with 70% or greater probabilities of exceeding the 10% seroprevalence threshold. We additionally classified areas where further data are needed to estimate serological exposure. These areas were classified as having exceedance probabilities between 40–60%, indicating further data are needed to determine whether these areas are above or below this threshold.

As fitting geostatistical models is likely not feasible for most control programmes, we compared estimates of seroprevalence derived from geostatistical models to more simple metrics of exposure which included the arithmetic mean, geometric mean, median, coefficient of variation and standard deviation of cluster MFI values. To evaluate these, we calculated the mean posterior estimates of seroprevalence for all households within each cluster from geostatistical models. Exploratory analysis identified the strongest correlation between arithmetic mean and seroprevalence. To further assess this metric, relationships between estimated seroprevalence and arithmetic mean MFI values per cluster were explored using B-spline regression, with optimal degrees selected using BIC.

## Results

A total of 10993 children from 201 clusters across 9 districts were sampled between November 2015 and April 2016. Data on 154 children were excluded due to high background or incomplete GPS data, resulting in a final dataset of 10840 children from 3444 households. The median age of children included was 5 years (IQR: 3–7), with roughly equal numbers of male and female children included (50.3% male, n = 5531). Distributions of antibody responses varied by pathogen (Fig A in S1 Text). Age-specific patterns of antibody responses were markedly different between antigens (Fig A in S1 Text). Antibody responses for VSP3, SEA and NIE all showed high responses in very young age groups, consistent with previous literature describing high exposure in very young children [15]. To further explore age-specific antibody responses, we compared densities between different age groups (Fig B in S1 Text). Using mixture models, we estimated seroprevalence and identified the distribution of MFI responses within the highest component (Table 1).

**Table 1:** Estimates of seroprevalence by antigen and disease, including distributions of responses for all individuals and individuals classified as exposed.

			MFI (median, IQR)	
Disease	Antigen	Exposed (p, %)	All individuals	Exposed
Trachoma	Pgp3	201 (1.9%)	10 (6–20)	9870 (2517–26115)
Lymphatic Filariasis	Wb123	54 (0.5%)	161 (79–326)	5688 (3633–9096)
Onchocerciasis	Ov16	116 (1.1%)	5 (3–7)	1002 (486–4294)
Strongyloidiasis	NIE	278 (2.6%)	43 (24–81)	865 (530–2192)
Schistosomiasis	SEA	1460 (13.5%)	275 (143–531)	1029 (871–1302)
Giardiasis	VSP3	967 (8.9%)	315 (115–1219)	15630 (10424–23933)
Yaws	rP17	19 (0.2%)	16 (11–25)	1842 (1208–20565)
	TmpA	8 (0.1%)	6 (4–9)	3722 (467–13088)

Using these data, we identified environmental and spatial risk factors for household seroprevalence of high responders for each disease. As the seroprevalence for yaws was very low, consistent with available data from this region [33], we excluded these antigens from spatial modelling. Final models identified different effects of covariates, with most diseases showing some association with climatic factors (Table 2). Varying spatial effects were observed across diseases; however, model fit was improved by the inclusion of spatial terms for all diseases.

**Table 2:** Mean posterior estimates of coefficients of fixed effects and spatial range, including 95% Bayesian credible intervals (BCI).

Covariate*	95% BCI		
	Mean	2.5%	97.5%
a. Trachoma Pgp3			
Annual precipitation (mm)	-0.568	-1.079	-0.066
Mean precipitation of warmest quarter (1970–2000), (mm)	0.182	-0.100	0.454
Mean precipitation of coldest quarter (1970–2000), (mm)	0.999	0.274	1.735
Mean temperature (1970–2000), (°C)	-1.225	-1.754	-0.709
Minimum temperature (1970–2000), (°C)	1.155	0.450	1.861
Accessibility, (hours to city)	0.106	-0.117	0.319
Soil type (water pH at 0cm)	0.252	-0.002	0.507
Spatial range (km)	17.416	8.016	34.97
b. Lymphatic filariasis Wb123			
Mean temperature (1970–2000), (°C)	-1.083	-1.692	-0.403
Minimum temperature (1970–2000), (°C)	1.008	0.303	1.601
Land surface temperature at night, (2002–2017), (°C)	-0.338	-0.739	0.036
Topographic wetness index	0.289	-0.085	0.637
Population density	-0.189	-0.696	0.254
Distance to roads (km)	0.176	-0.096	0.413
Soil type (water pH at 0cm)	0.776	0.291	1.255
Spatial range (km)	43.01	8.852	141.62
c. Onchocerciasis Ov16			
Annual precipitation (mm)	-0.348	-0.833	0.147
Land surface temperature at night, (2002–2017), (°C)	-0.686	-1.046	-0.344
Topographic wetness index	-0.343	-0.721	0.013
Accessibility, (hours to city)	0.501	0.010	0.978
Soil type (proportion of silt at 0cm)	0.310	-0.040	0.666
Spatial range (km)	29.83	14.67	55.17
d. Schistosomiasis SEA			
Mean precipitation of warmest quarter (1970–2000), (mm)	0.528	0.347	0.710
Mean precipitation of coldest quarter (1970–2000), (mm)	0.619	0.439	0.800
Mean temperature, (1970–2000), (°C)	0.620	0.440	0.799
Distance to water bodies, (km) Normalised difference vegetation index	-0.167 0.214	-0.347 0.104	0.012 0.325
Distance to roads, (km)	-0.381	-0.564	-0.197
Spatial range (km)	7.102	6.420	8.054
e. Strongyloidiasis NIE			
Annual precipitation (mm)	0.326	0.037	0.615
Mean precipitation of warmest quarter (1970–2000), (mm)	-0.245	-0.525	0.035
Mean precipitation of coldest quarter (1970–2000), (mm)	-0.575	-0.890	-0.260
Minimum temperature, (1970–2000), (°C)	-0.222	-0.506	0.061
Insecticide treated net coverage Population density	0.213 -0.100	-0.062 -0.209	0.488 0.009
Soil type (silt at 0cm)	-0.158	-0.367	0.050
Distance from roads (km)	0.349	-0.059	0.757
Spatial range (km)	26.235	3.458	98.559
f. Giardia VSP3			
Annual precipitation (mm)	0.113	-0.032	0.257
Mean precipitation of warmest quarter (1970–2000), (mm)	-0.180	-0.304	-0.056
Mean precipitation of coldest quarter (1970–2000), (mm)	-0.332	-0.498	-0.166
Land surface temperature at night, (2002–2017), (°C)	-0.107	-0.211	-0.003
Minimum temperature, (1970–2000), (°C) Normalised difference vegetation index	-0.137 -0.111	-0.288 -0.200	0.013 -0.021
Spatial range (km)	12.315	2.880	33.69

* All covariates mean-centred and scaled

**Fig 2:**
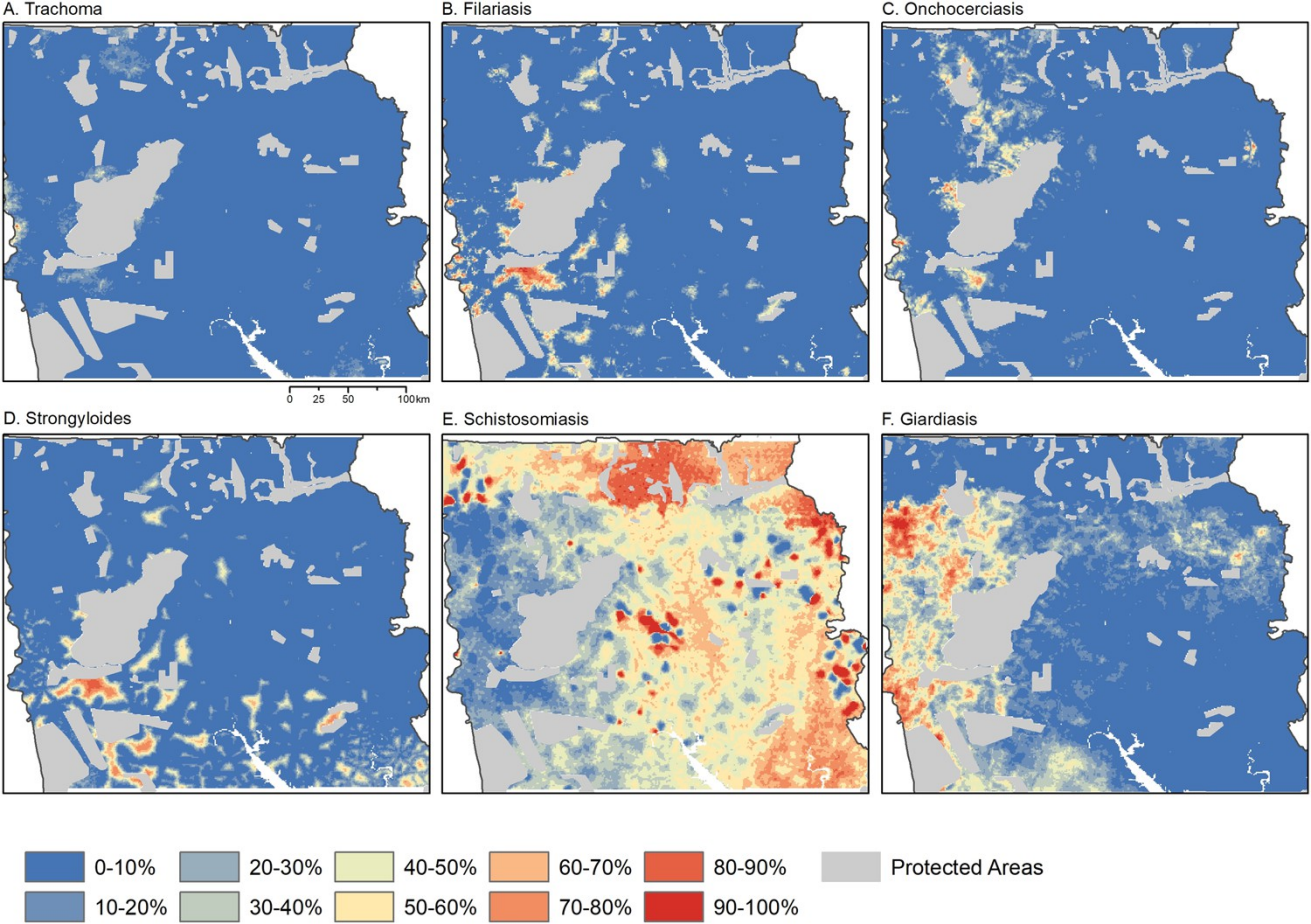
Posterior estimates of the probability of exceeding 10% seroprevalence of high responders to A) Trachoma Pgp3; B) Lymphatic filariasis Wb123; C) Onchoceriasis Ov16; D) Strongyloidiasis NIE; E) Schistosomiasis SEA; F) Giardiasis VSP3; administrative shapefiles obtained from National Information Technology Agency (NITA), Government of Ghana (https://data.gov.gh/dataset/shapefiles-all-districts-ghana-170-districts).

**Fig 3:**
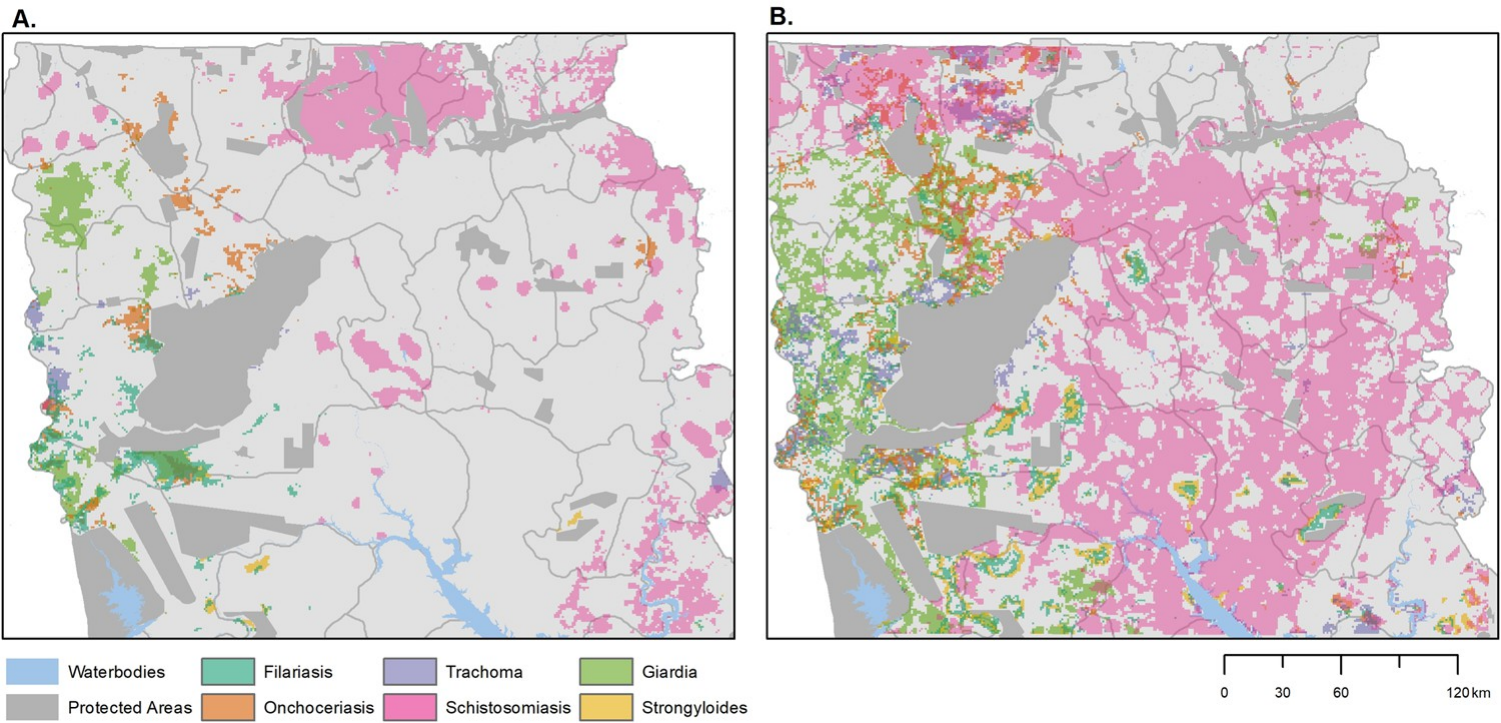
Combining disease measures to A) identify regions with high probabilities of exceeding 10% seroprevalence (exceedance probabilities > 70%); and B) identify regions with high uncertainty (exceedance probabilities 40–60%); administrative shapefiles obtained from National Information Technology Agency (NITA), Government of Ghana (https://data.gov.gh/dataset/shapefiles-all-districts-ghana-170-districts).

Within Northern Ghana, we predicted the probability of exceeding the 10% seropositivity at 1000m resolution (Fig 2). Using these exceedance probabilities, we identified regions with high probabilities of recent transmission of multiple diseases (Fig 3A). We also found areas with high probabilities of multiple diseases, such as at the western border of the study area; this may indicate poor coverage by health programmes, human movement from neighbouring countries or other risk factors. We additionally identified priority areas for future surveys based on high uncertainty; these were defined as having exceedance probabilities between 40 and 60%, indicating models were not able to determine whether these regions were above or below 10% seropositivity (Fig 3B).

**Fig 4:**
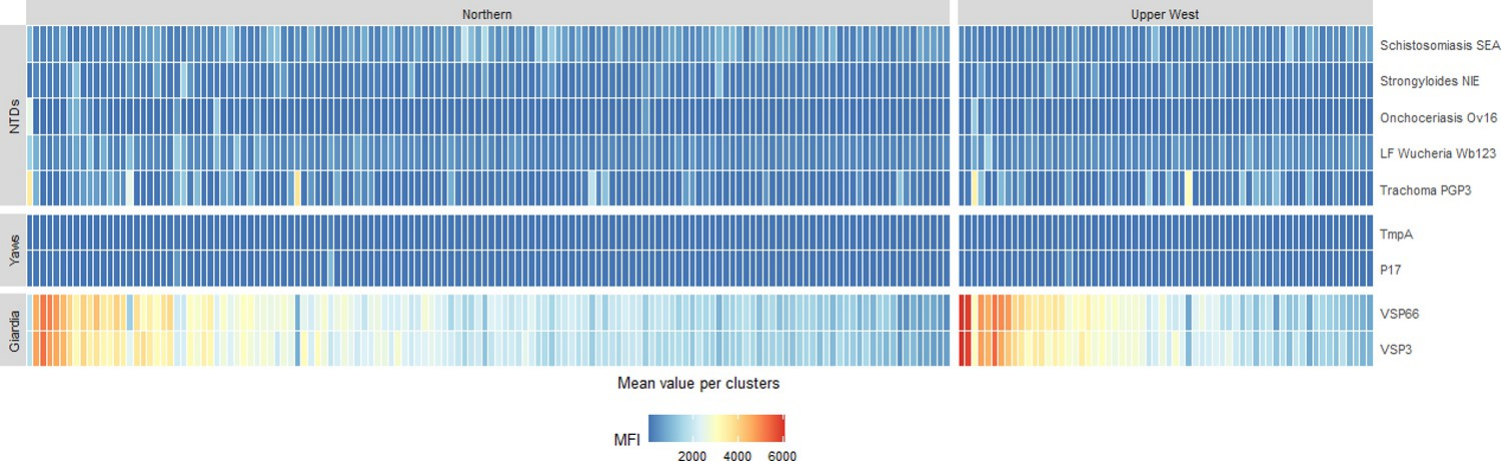
Arithmetic mean MFI values per cluster by region.

When simple metrics of cluster MFI values were compared to the exceedance probabilities for all households within the cluster, the arithmetic mean MFI showed the strongest correlation with mean posterior estimates of cluster seroprevalence. Heterogeneity between MFI values allowed identification of high-risk clusters (Fig 4). Relationships between these metrics were nonlinear and highly variable for all diseases surveyed, with arithmetic mean MFI values closely associated with cluster-level seroprevalence for high burden diseases (e.g. schistosomiasis) but less correlated for lower burden diseases (Fig C in S1 Text). However, for all diseases surveyed, prioritising clusters with the highest mean MFI values would enable targeting clusters with the highest probabilities of recent exposure.

## Discussion

This study demonstrates the utility of geostatistical modelling of integrated serological surveys to characterise spatial patterns of NTDs and other pathogens. Despite the increasing use of this MBA, to our knowledge, this is the first study integrating multi-disease data within geostatistical frameworks. By estimating proportions of individuals with elevated antibody levels suggesting recent exposure, we demonstrate how serological data can be combined with contemporaneous spatial and environmental data. These geostatistical models of serological data broadly reflect known disease status, while additionally highlighting some previously unknown foci of possible transmission. This could become a valuable tool for control programmes operating in low transmission settings where probabilities of detecting infections are extremely low. In these cases, analysis of serological data within geostatistical frameworks enables identification of environmental and spatial risk factors associated with residual transmission and allows prioritisation of areas for future control and surveillance efforts. Further, we demonstrate how simple metrics of antibody responses can provide actionable information to control and elimination programmes to prioritise areas with high probabilities of transmission.

Although a large body of literature explores the utility of serological assays as measures of transmission, these data are rarely analysed within geostatistical frameworks and then primarily for single diseases (e.g. [21,46]). This is in part due to the challenges linking serological data, which may represent past exposure, with spatial and environmental data representing current locations and conditions. While in some cases, simple maps of serological data may be sufficient to delineate the boundaries of transmission or monitor infection introductions, identification of areas of on-going transmission of a previously endemic disease requires estimation of recent exposure. There remains no standard approach for classifying seropositivity from these MBAs, with reference values typically only available for vaccine preventable diseases [19]. Similar to previous studies, we identified very high rates of exposure to entero-pathogens in young children, necessitating fitting mixture models using subsets of the youngest children to ensure sufficient unexposed individuals [15]. In the absence of longitudinal data on antibody kinetics, identifying high responses can act as a proxy for recent or repeated exposure. Within this study, a three-component mixture model was used to identify the highest responders for each antigen. While this has the advantage of identifying individuals with the highest antibody titres and priorities for future surveillance, estimates of seropositivity should be interpreted cautiously as this may not represent all exposed individuals. Similarly, public health implications of groups identified with high responses may vary by disease and epidemiology within this context. If more detailed longitudinal measurements were available, future studies could explore mechanistic modelling of antibody dynamics to more accurately identify exposure groups (e.g. [47,48]). However, the correlation between serological estimates and other metrics of disease transmission strongly supports the use of these assumptions.

Within this setting, serological data support other estimates of NTD burdens and associated environmental risk factors within Northern Ghana. These results are consistent with previous findings of very low prevalence of trachoma and additionally demonstrate how serology may be employed to identify areas for post-elimination surveillance for potential recrudescence [27,28]. Additionally, analysis of serological data on exposure to endemic pathogens can be used to prioritise areas with high probabilities of recent transmission or high uncertainty for one or multiple diseases, to identify targets for future control or surveillance efforts. In particular, examination of areas with high uncertainty reveals key knowledge gaps. For example, despite strong associations with distance from water and other environmental predictors, household-level prevalence of schistosomiasis is unknown for most of the study sites due to the extremely high focality of the disease. These levels of focality may not be represented by school-based or other indicator surveys not capturing the full distribution of schistosomiasis within the community. The uncertainty in model results also reflects the distribution of sampling points; visualisation of this uncertainty allows prioritisation of areas for future surveys. This has important implications both when planning surveillance and when allocating treatment at larger administrative units, as is commonly done for mass drug administration [49].

Geostatistical modelling of serological data representing exposure to multiple pathogens has important implications for survey design. The systematic and random population-based sampling strategy used for trachoma mapping enabled assessment of the distribution of other NTDs; this approach would not have been feasible with purposive sampling. For example, this study identified a focus of onchocerciasis with a significant prevalence of anti-Ov16 antibodies in children aged 1–9 years in Saboba-Cherepon. Previous mapping of onchocerciasis in this area through the Rapid Epidemiological Mapping of Onchocerciasis (REMO) in 2009 determined this to be an area of hypo-endemicity, not requiring onchocerciasis specific MDA at that time. Since then, the area has been treated for lymphatic filariasis, which includes the delivery of ivermectin (the drug also given in MDA for onchocerciasis) and the expectation had been this would also impact on *O*. *volvulus* transmission. However, lymphatic filariasis MDA ceased in 2014, and these serological data indicate an increase in onchocerciasis previously unknown to GHS and as a direct result of the integrated surveillance approach, GHS decided to start MDA specifically for onchocerciasis in this focus (personal communication, Ghana Health Service). While further work is required to confirm transmission and disease burdens may have changed since 2015, this identifies a priority area for surveillance.

For control and elimination programmes needing to make operational decisions quickly without access to technical expertise needed to develop geostatistical models, very simple metrics can be applied to identify locations at high risk of transmission. While the relationship between arithmetic mean MFI values and estimated seroprevalence is not linear, prioritising control activities at clusters with the highest mean MFI values would likely ensure that interventions are reaching communities with the highest seroprevalence. This agrees with previous studies showing high correlation between mean quantitative antibody levels and other infection-based metrics of NTD transmission [14,24]. As serological samples can be collected using finger-prick blood sampling with no need for cold chains in the field, this is an operationally attractive and cost-effective method compared to many other diagnostics. This utility is further increased by the ability to multiplex, allowing information to be collected for multiple diseases of public health importance [19,50]. Ideally, future studies could compare metrics obtained by integrated serological surveys with routinely used diagnostics to enable more efficient application for disease mapping surveys and treatment decisions.

Despite the value of this approach, this study had several important limitations. As this study does not include adults, further validation of these methods may be required in settings with very high historical transmission and exposure in older age groups. While serological surveys of children are likely to be a better marker of recent rather than historical exposure, the utility of this approach will vary for specific diseases and associated risk factors. For trachoma, young children under 9 are believed to be the primary source of infection. In contrast, children aged under 5 years have lower risks of onchocerciasis and schistosomiasis, which predominantly impact school-aged children and adults with high-risk occupational activities. This highlights a key methodological challenge of integrated surveys targeting multiple diseases with different risk groups and transmission mechanisms. Additionally, this analysis relied on a single dataset collected over one time point. These diseases may additionally have differing immune responses, antibody kinetics and infection periods and longitudinal data may be able to more accurately identify recent infections or characterise transmission [48]. The uncertainty identified within these models may be further reduced by inclusion of other data and surveillance information and cannot be considered a reflection of the current knowledge of disease control across this region. However, the Bayesian framework used can be easily extended to incorporate other sources of information, such as pairing serological data with infection data [51].

Despite these limitations, this study demonstrates how integrated serological surveillance can characterise the spatial distribution of exposure to multiple pathogens. An adaptable framework is provided to understand the spatial and environmental factors driving transmission in elimination settings when infection data are rare. As countries approach elimination, this study additionally highlights the need for innovative surveillance approaches utilising population representative sampling to maximising efficiency by collecting data across multiple diseases. Applying these techniques can provide valuable information for control programmes needing to identify and target remaining foci of infection.

## Supporting information

S1 Text Supplementary Information file. **Table A.** Priors used for proportions in three component mixture models. **Table B.** Spatial and environmental covariates. **Fig A.** Disease-specific antibody responses for all included children, i. Density plots of log-transformed MFI values, ii. Age distributed antibody responses. **Fig B.** Antibody densities by age categories. **Fig C.** Relationships between mean posterior estimates of seroprevalence and arithmetic mean MFI per cluster for: A) Trachoma; B) Filariasis; C) Onchocerciasis; D) Strongyloides; E) Schistosomiasis; F) Giardiasis.
(DOCX)
