# Supplementary material for: Characterising spatial patterns of neglected tropical disease transmission using integrated sero-surveillance in Northern Ghana
Source: PLoS Negl Trop Dis. 2022 Mar 8;16(3):e0010227. doi: 10.1371/journal.pntd.0010227 (PMC8932554; doi:10.1371/journal.pntd.0010227)
Supplement: S1 Text — Table A. Priors used for proportions in three component mixture models. Table B. Spatial and environmental covariates. Fig A. Disease-specific antibody responses for all included children, i. Density plots of log-transformed MFI values, ii. Age distributed antibody responses. Fig B. Antibody densities by age categories. Fig C. Relationships between mean posterior estimates of seroprevalence and arithmetic mean MFI per cluster for: A) Trachoma; B) Filariasis; C) Onchocerciasis; D) Strongyloides; E) Schistosomiasis; F) Giardiasis. (DOCX) [file pntd.0010227.s001.docx]

**Supplementary Information: Characterising spatial patterns of food-borne, water-borne, and neglected tropical disease transmission using integrated sero-surveillance in Northern Ghana**

**Running title:** Integrated sero-surveillance in Ghana

**Authors:** Kimberly Fornace^1^*, Laura Senyonjo^2^, Diana L. Martin^3^, Sarah Gwyn^3^, Elena Schmidt^2^, David Agyemang^4^, Benjamin Marfo^5^, James Addy^5^, Ernest Mensah^6^, Anthony W. Solomon^1,7^, Robin Bailey^1^, Chris Drakeley^1^, Rachel Pullan^1^

**Table A.** Priors used for proportions in three component mixture models

| Disease | Unexposed (1) | Historical (2) | Recent/ High (3) |
| --- | --- | --- | --- |
| Trachoma [1] | 0.70 | 0.20 | 0.10 |
| Yaws [2] | 0.95 | 0.04 | 0.01 |
| Lymphatic filariasis [3, 4] | 0.70 | 0.20 | 0.10 |
| Onchocerciasis [4] | 0.70 | 0.20 | 0.10 |
| Strongyloidiasis [4] | 0.50 | 0.40 | 0.10 |
| Schistosomiasis [4]  Giardiasis* [5] | 0.50  0.30 | 0.30  0.30 | 0.20  0.40 |

* No local data available; assumed to be high based on seroprevalence surveys from similar settings

**Table B.** Spatial and environmental covariates

| Variable | Spatial Resolution | Year |
| --- | --- | --- |
| Population density | 100m | 2015 |
| Accessibility to nearest city, modelled as travel time in minutes to nearest urban centre | 1000m | 2015 |
| Insecticide treated net coverage | 1000m | 2015 |
| Euclidean distance to roads | 30m | N/A |
| Euclidean distance to water bodies | 30m | N/A |
| Elevation | 250m | 2015 |
| Topographic Wetness Index | 250m | 2015 |
| Normalised difference vegetation index | 30m | 2015 – 2016 |
| Normalised difference water index | 30m | 2015 – 2016 |
| Forest cover | 30m | 2015 |
| Clay fractions (0, 5, 15 cm) | 250m | N/A |
| pH water (0, 5, 15 cm) | 250m | N/A |
| Sand fraction (0, 5, 15 cm) | 250m | N/A |
| Silt fraction (0, 5, 15 cm) | 250m | N/A |
| Land surface temperature day | 1000m | Average from 2002- 2017 |
| Land surface temperature night | 1000m | Average from 2002 - 2017 |
| Annual precipitation | 1000m | Average from 2002 - 2017 |
| Evapotranspiration | 1000m | Average from 2002 - 2017 |
| Aridity index | 1000m | Average from 2002 - 2017 |
| Bioclimatic variables, including annual mean temperature, mean diurnal range, isothermality (mean diurnal range/ annual temperature range), temperature seasonality (standard deviation), maximum temperature of the warmest month, minimum temperature of the coldest month, temperature annual range, mean temperature of the wettest quarter, mean temperature of the driest quarter, mean temperature of the warmest quarter, mean temperature of the coldest quarter, annual precipitation, precipitation of the wettest month, precipitation of the driest month, precipitation seasonality (coefficient of variation), precipitation of the wettest quarter, precipitation of the warmest quarter, precipitation of the coldest quarter | 1000m | 1970 – 2000 climate metrics |

**Fig A.** Disease-specific antibody responses for all included children, i. Density plots of log-transformed MFI values, ii. Age distributed antibody responses

i.


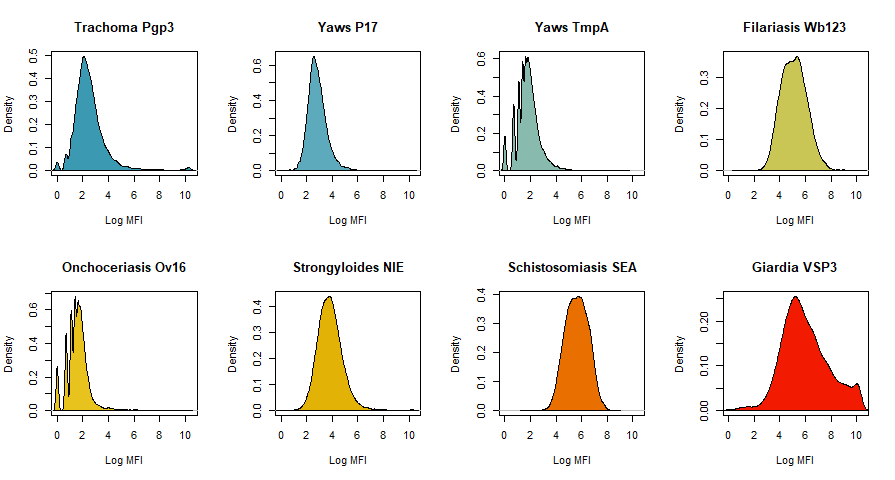


ii.


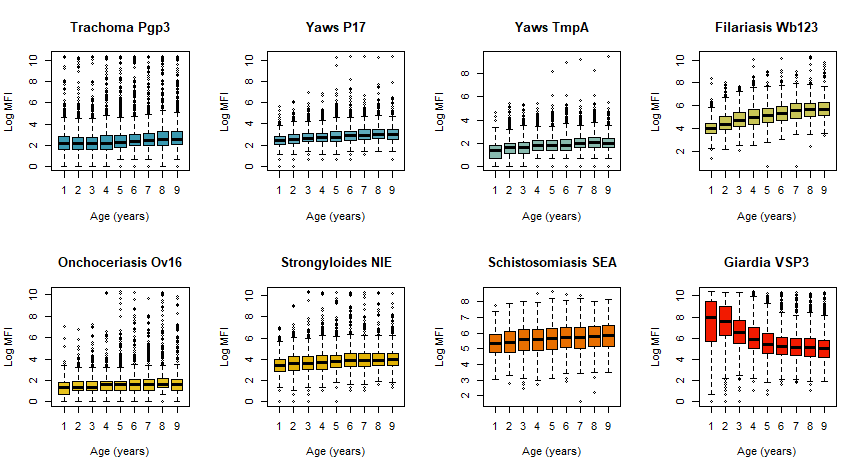


**Fig B.** Antibody densities by age categories


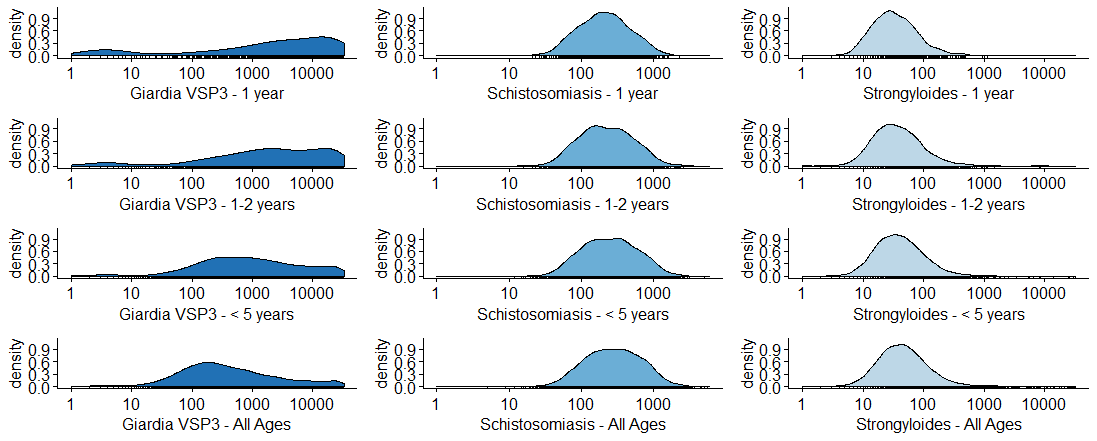


**Fig C.** Relationships between mean posterior estimates of seroprevalence and arithmetic mean MFI per cluster for: A) Trachoma; B) Filariasis; C) Onchocerciasis; D) Strongyloides; E) Schistosomiasis; F) Giardiasis


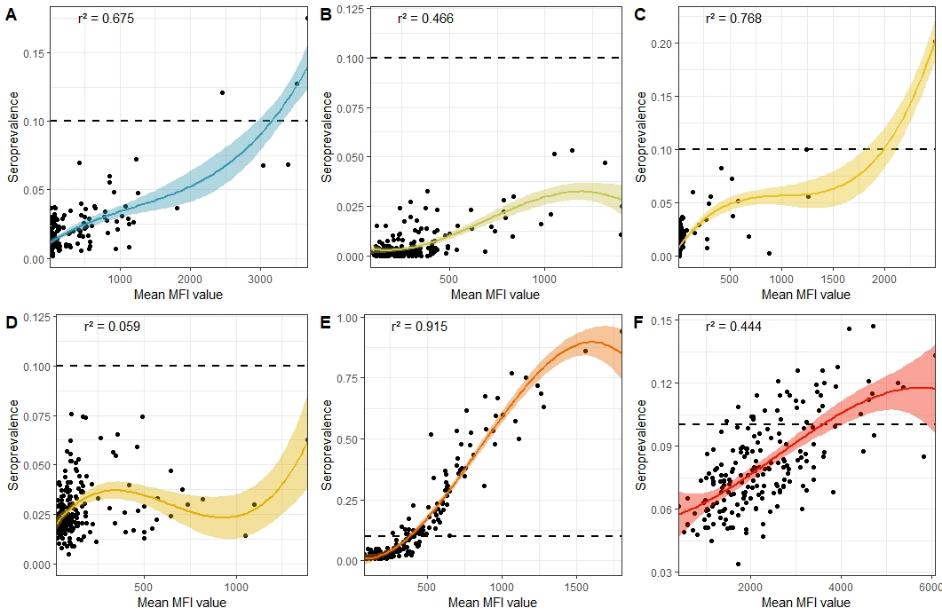


References

1. Senyonjo, L.G., et al., *Serological and PCR-based markers of ocular Chlamydia trachomatis transmission in northern Ghana after elimination of trachoma as a public health problem.* PLoS Negl Trop Dis, 2018. **12**(12): p. e0007027.

2. Ghinai, R., et al., *A cross-sectional study of 'yaws' in districts of Ghana which have previously undertaken azithromycin mass drug administration for trachoma control.* PLoS Negl Trop Dis, 2015. **9**(1): p. e0003496.

3. Aboagye-Antwi, F., et al., *Transmission indices and microfilariae prevalence in human population prior to mass drug administration with ivermectin and albendazole in the Gomoa District of Ghana.* Parasit Vectors, 2015. **8**: p. 562.

4. ESPEN, *Expanded Special Project for Elimination of Neglected Tropical Diseases: Ghana*, W.H.O.R.O.f. Africa, Editor. 2020: Brazzavile.

5. Arnold, B.F., et al., *Enteropathogen antibody dynamics and force of infection among children in low-resource settings.* Elife, 2019. **8**.
